# 1280. Real-World Comparison of HIV-ASSIST with Expert Opinion in Selecting Antiretroviral Therapy for Complex Patients

**DOI:** 10.1093/ofid/ofac492.1111

**Published:** 2022-12-15

**Authors:** Michael E Tang, Lucas Hill, Jeffrey Yin, Kari Abulhosn, Darcy Wooten

**Affiliations:** University of California, San Diego, San Diego, California; University of California, San Diego, San Diego, California; University of California, San Diego, San Diego, California; UCSD, San Diego, California; University of California, San Diego, San Diego, California

## Abstract

**Background:**

HIV-ASSIST is an online, clinical decision support tool that helps HIV clinicians select antiretroviral (ARV) regimens for patients with HIV by accounting for individual patient characteristics. Concordance between HIV-ASSIST recommendations and expert opinion has been reported to be as high as 89% in treatment-experienced patients. We evaluated the utility of the HIV-ASSIST tool for a heavily treatment-experienced, complex patient population at the University of California, San Diego (UCSD) by comparing regimens recommended by HIV-ASSIST with regimens recommended by HIV experts.

**Methods:**

We identified 14 patients through a routine HIV drug resistance teaching conference at our clinic. Each case was reviewed by 5 HIV clinical experts who independently recommended an ARV regimen. A consensus “best” regimen was agreed upon among the 5 experts for each patient. Consensus regimens were compared for concordance to the top 5 regimens recommended by the HIV-ASSIST tool. HIV-ASSIST regimens were also reviewed to determine if any were high or moderate risk for virologic failure due to patient or resistance characteristics, or drug-drug interactions (DDIs).

**Results:**

The patients analyzed were medically and psychosocially complex, with a high rate of multi-class resistance (Table 1). Expert-recommended regimens were concordant with one of the top five regimens recommended by the HIV-ASSIST tool for 4/14 (28%) patients (Table 2). We further classified 20/70 (29%) regimens as high risk for virologic failure and 12/70 (17%) regimens as moderate risk for virologic failure (Figure 1).

**Figure:**
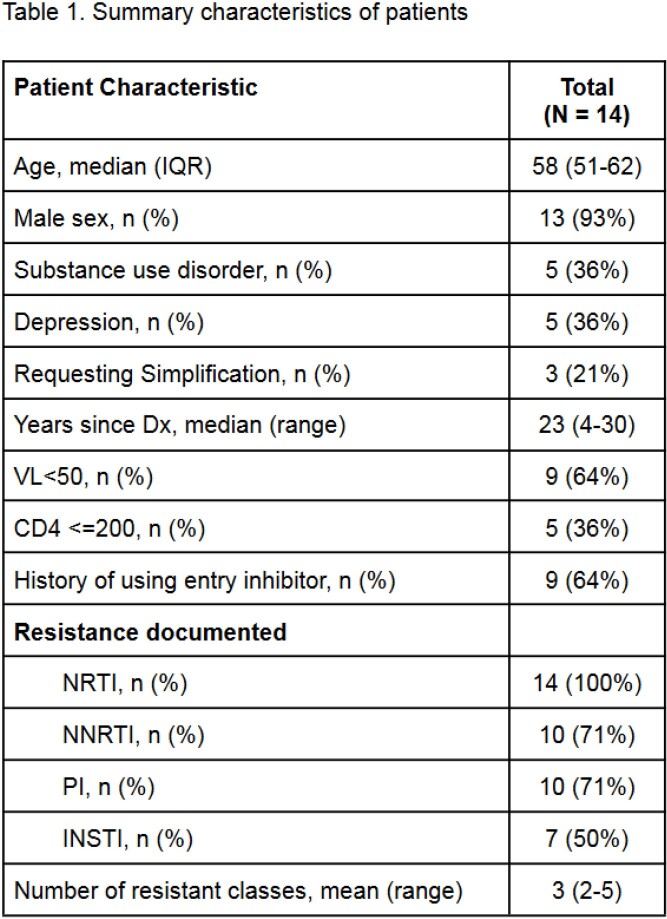

**Table 2.**
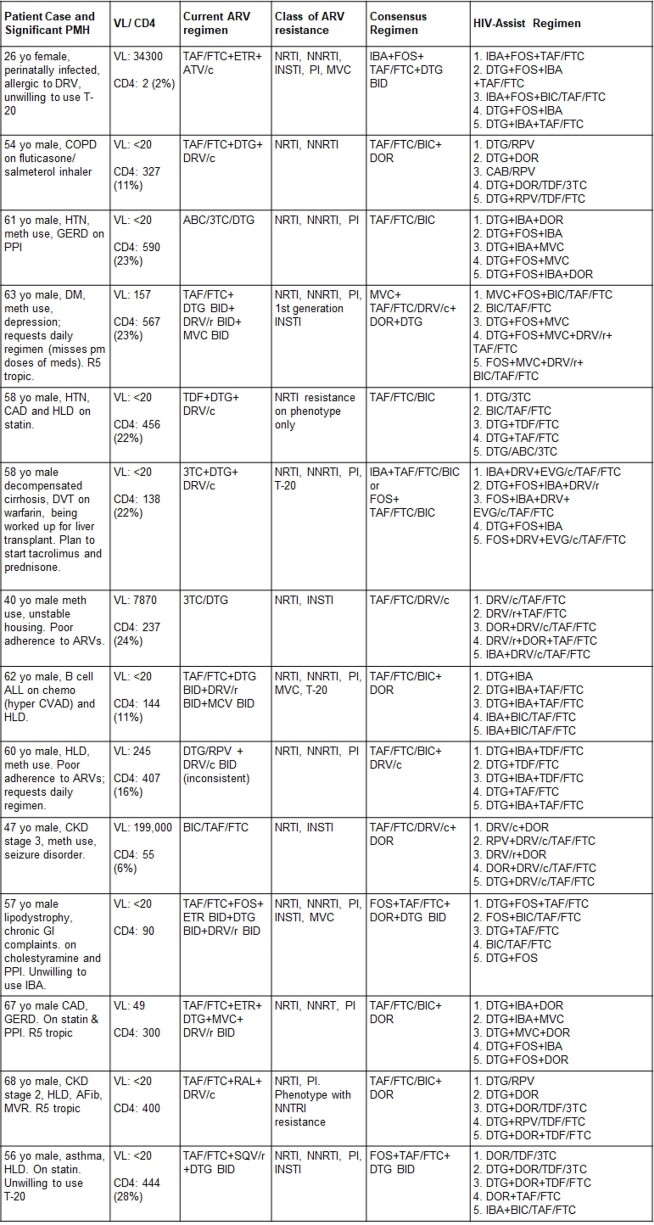
Expert-recommended consensus regimens compared to the top 5 HIV-ASSIST regimens for 14 patients with HIV and a history of drug resistance, multimorbidity, and polypharmacy.

**Figure 1:**
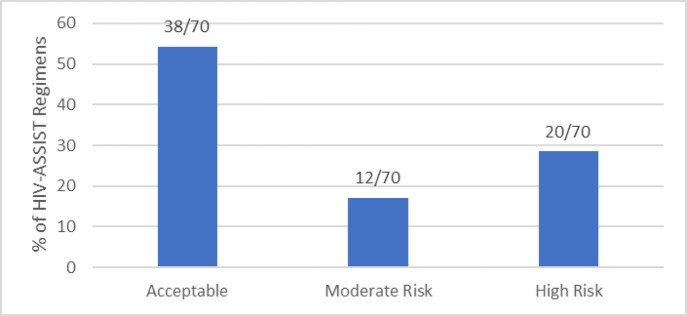
Classification of HIV-ASSIST regimens by HIV clinical experts as either acceptable, moderate risk or high risk for virologic failure.

**Conclusion:**

Compared to prior reports, we found lower concordance between ART regimens recommended by HIV experts vs those recommended by the HIV-ASSIST tool in patients with HIV drug-resistance and/or complex comorbidities and potential DDIs. Moreover, several HIV-ASSIST regimens were considered at risk for virologic failure. We recommend caution in using the HIV-ASSIST tool for complex patients with significant drug resistance.

**Disclosures:**

**Lucas Hill, PharmD, AAHIVP**, Gilead Sciences: Speakers Bureau.

